# A Novel Test for Gene-Ancestry Interactions in Genome-Wide Association Data

**DOI:** 10.1371/journal.pone.0048687

**Published:** 2012-12-06

**Authors:** Joanna L. Davies, Jean-Baptiste Cazier, Malcolm G. Dunlop, Richard S. Houlston, Ian P. Tomlinson, Chris C. Holmes

**Affiliations:** 1 Department of Statistics, University of Oxford, Oxford, United Kingdom; 2 Wellcome Trust Centre for Human Genetics, University of Oxford, Oxford, United Kingdom; 3 Colon Cancer Genetics Group, Institute of Genetics and Molecular Medicine, University of Edinburgh and Medical Research Council Human Genetics Unit, Western General Hospital, Edinburgh, Scotland, United Kingdom; 4 Section of Cancer Genetics, Institute of Cancer Research, Sutton, Surrey, United Kingdom; 5 Oxford Comprehensive Biomedical Research Centre, John Radcliffe Hospital, Headington, Oxford, United Kingdom; 6 MRC Harwell, Harwell Science and Innovation Campus, Oxfordshire, United Kingdom; Democritus University of Thrace, Greece

## Abstract

Genome-wide association study (GWAS) data on a disease are increasingly available from multiple related populations. In this scenario, meta-analyses can improve power to detect homogeneous genetic associations, but if there exist ancestry-specific effects, via interactions on genetic background or with a causal effect that co-varies with genetic background, then these will typically be obscured. To address this issue, we have developed a robust statistical method for detecting susceptibility gene-ancestry interactions in multi-cohort GWAS based on closely-related populations. We use the leading principal components of the empirical genotype matrix to cluster individuals into “ancestry groups” and then look for evidence of heterogeneous genetic associations with disease or other trait across these clusters. Robustness is improved when there are multiple cohorts, as the signal from true gene-ancestry interactions can then be distinguished from gene-collection artefacts by comparing the observed interaction effect sizes in collection groups relative to ancestry groups. When applied to colorectal cancer, we identified a missense polymorphism in iron-absorption gene *CYBRD1* that associated with disease in individuals of English, but not Scottish, ancestry. The association replicated in two additional, independently-collected data sets. Our method can be used to detect associations between genetic variants and disease that have been obscured by population genetic heterogeneity. It can be readily extended to the identification of genetic interactions on other covariates such as measured environmental exposures. We envisage our methodology being of particular interest to researchers with existing GWAS data, as ancestry groups can be easily defined and thus tested for interactions.

## Introduction

Genome-wide association studies have had a marked impact on our understanding of the contribution of common variants to common disease [1], [2]. The prevalence of common diseases often varies across ethnic groups [3], and hence it is perhaps not too surprising to observe systematic differences in marker effect sizes for GWAS in more closely related populations [4]–[6]. A fundamental difficulty in testing whether such differences arise owing to chance, study design and execution, or true population-specific effects, is that the sample collection label is typically confounded with the interaction effect.

To circumvent these problems, we have developed a robust statistical test for gene-ancestry interactions that makes use of observed and measured relatedness between closely-related populations. The method re-assigns individuals to new ancestry groups by clustering in the space defined by the leading principal components of the empirical genotype matrix [7]–[10] and then tests for differences in disease association signal between these new ancestry groups. We then compare this evidence with analogous tests for disease association performed with the original collection labels. When populations are closely related, we expect overlap between the collections in the principal component space such that the allocation of samples to ancestry groups differs from that of the original collection labels. If heterogeneity is observed and is due to genetic background (or a variable correlated with genetic background), the effect size and signal of heterogeneity should increase when individuals are re-grouped by relatedness, whereas for collection artefacts the effect size and signal of heterogeneity should decrease. Signals of genetic heterogeneity observed by chance can be filtered out in a replication stage by repeated testing in multiple independent sample sets, even if these lack the necessary information for ancestry assignment.

Since intra-study effect heterogeneity can obscure true associations between polymorphisms and disease, a potential benefit of our approach is the un-masking of true associations that are specific to ancestral groups or stronger in one group than another. Although intra-study gene-gene interactions or gene-pathway interactions have previously been analysed [11], [12] no study has previously addressed GWAS gene-ancestry interactions.

## Results

### Overview of the gene-ancestry interaction method

**Figure 1:**
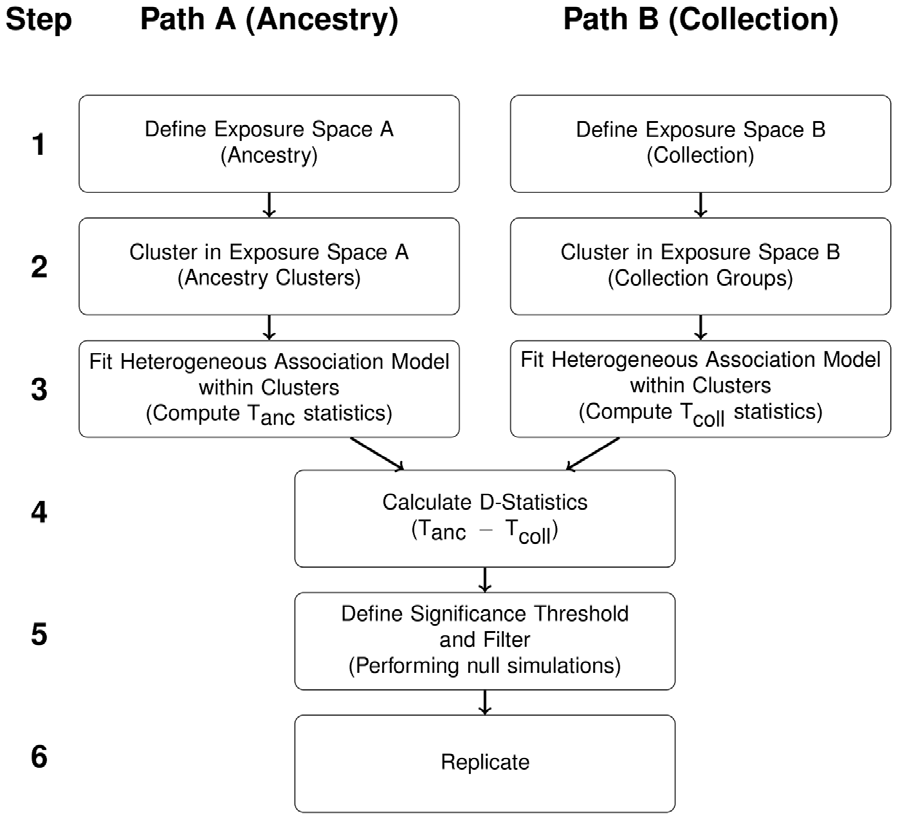
Summary of the Methodology. Flow diagram showing methodological steps for testing for interactions when there are two comparable sources of heterogeneity. There are two paths (A and B) in the work flow, one for each source. In our case path A corresponds to ancestry and path B corresponds to collection. If there is only a single collection, path A can be followed with steps 1A, 2A, 3A, 5, and 6 (i.e. without computation of D-Statistics). T_anc_ is the heterogeneity test statistic computed with the ancestry clustering and T_coll_ is the heterogeneity test statistic computed with the collection clustering as defined in the methods.

The method is summarised in Figure 1 and details are provided in the *Materials and methods* section. In brief, the analysis pipeline consists of an initial step where two or more GWAS have been identified on the same disease or trait of interest across closely related populations. SNPs are then identified which appear to show an interaction on ancestry; by first clustering the combined set of individuals into ancestry groups and then statistically testing for evidence of an ancestry group interaction term of association with the trait. SNPs identified as putative interaction markers are then tested for strength of association for an interaction on the original cohort groups. For true ancestry-interactions the effect size should increase when clustering on ancestry rather than cohort label. Markers are then filtered to take forward only those showing largest difference. Markers for which the statistical evidence of an interaction is overwhelming, or ones showing milder evidence but with additional functional or biological relevance linking the marker to the trait, are taken forward to a Replication stage. In the replication stage it is valid to only genotype the marker(s) of interest in individuals sampled from the original population groups. In this situation you are not abel to assign such replication individuals to ancestry groups, but the population label can be used as a noise proxy without bias, but with some loss in power. Below, we present our results for a GWAS of colorectal cancer.

### Application of the method to a GWAS of colorectal cancer susceptibility

#### Discovery Stage

The discovery stage involves steps 1 to 5 in Figure 1. To illustrate the method and test its effectiveness, we initially analysed data from 13,690 individuals obtained from two parallel colorectal cancer (CRC) GWAS studies performed in England and Scotland. The data has previously been analysed and reported using conventional GWAS methods without consideration of ancestry specific effects [13]–[16]. In the original study there were two “Phases” of data collection. In Phase 1 an initial genome-wide scan in 3,831 individuals - 1,849 from England and 1,982 from Scotland - genotyped using the Illumina Hap550 or equivalent array, followed by a more focussed Phase 2 whereby 40,892 SNPs showing the best evidence of association in Phase 1 were genotyped in an independent set of 9,859 individuals, comprising 5,744 from England and 4,115 from Scotland. Since both Phases 1 and 2 of the data were available to us from the outset, we used both of these for the discovery stage of our analysis. It is important to note that for most studies we envisage only having available a single Phase of discovery data; in which case the below would be modified accordingly.

**Figure 2:**
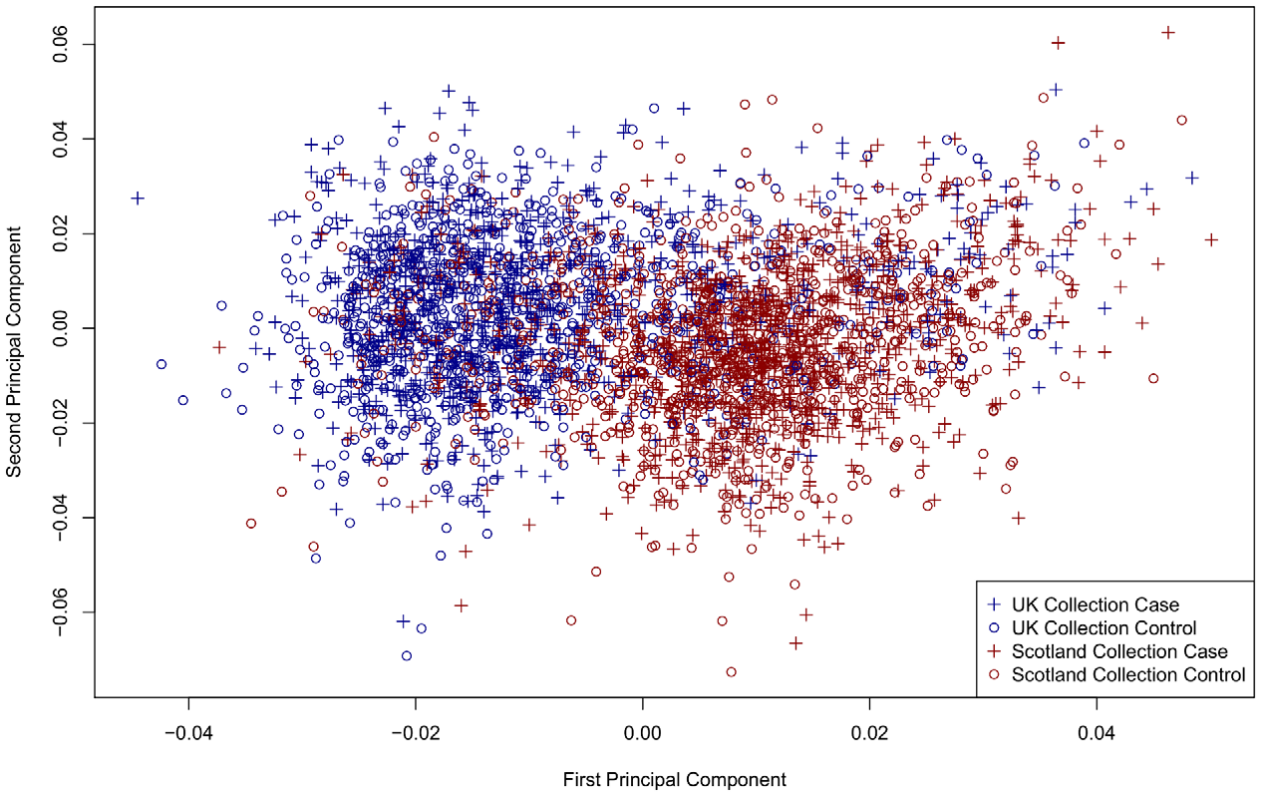
First PC versus Second PC in Phase 1. Individuals in Phase 1 plotted in the space defined by the first two PCs computed from the genotype matrix. Individuals are coloured by their collection group and case-control status indicated by the plotting symbol. There is considerable overlap between the collection groups.

We characterised genetic ancestry by performing a principal component analysis (PCA) after filtering out SNPs in regions of long-range linkage disequilibrium [17] and pruning remaining markers using a short-range LD filter (see *Materials and methods*). In Phase 1, only the first two principal components (PCs) provided a significant measure of separation between the two groups collected from England and Scotland (Figure 2) and we used these two components to group individuals according to their empirical relatedness using the k-means clustering algorithm [18]. We assessed a range of k values by computing the Bayesian Information Criterion (BIC) [19] for each. The optimised BIC was with k = 2 and we refer to the resulting two clusters as the English and the Scottish “ancestry groups”, in contrast to the original English and Scottish “collection groups” (Figure 2). Clustering by ancestry resulted in 18.5% of individuals changing label - that is, these individuals were originally registered in, say, the Scottish collection group, but were assigned to the English ancestry group after clustering, or vice versa (see Table S2).

We then looked for single marker-ancestry interactions genome-wide by fitting the heterogeneous logistic model of association (that is, detecting different genetic associations with disease for different ancestry groups) and comparing the fit of this model to that of the null model with the same genetic association across all clusters (although allowing distinct intercept terms for each cluster). Since the first principal genetic component used to characterise ancestry was disease-associated, we included this together with gender as main effect adjustment covariates in both the heterogeneous and null models. To compare the model fits, we used the BIC which adjusts the model fit for complexity. We calculated the difference in BIC statistics between the null-BIC and heterogeneous-BIC models and used this difference as a test statistic (denoted 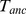, see *Materials and methods*).

We also performed an analogous scan for single marker-collection interactions by using the original collection groups in place of ancestry, “Path B” in Figure 1, thus computing a new set of test statistics (denoted 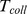). 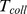 statistics are analogous to 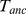 statistics, but computed using models fitted within collection groups rather than ancestry groups. True heterogeneity on genetic background should result in an increased signal of interaction association (that is, 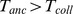), whereas collection artefacts will tend to have 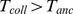. From this, we highlighted for each SNP the difference 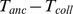, referred to as the “D-statistic”(see *Materials and methods*). We retained markers above the 1% D-statistic significance threshold (Figure S3), estimated by simulation under the null hypothesis of no disease association for any individual (see *Materials and methods*). After imposing this filter, 4,445 out of 485,757 markers remained, consistent with the number (4,857) expected by chance.

In our special case we also have discovery data on a second Phase. We then made use of the Phase 2 data in our discovery stage by performing the same heterogeneity analysis (steps 1 to 4 in Figure 1) and looking for signals of heterogeneity consistent with those observed in Phase 1. We found that 388 of the 4,445 markers identified for Phase 1 had been genotyped in Phase 2 of the original study. We followed the same procedure used in Phase 1 by running smartpca on the combined Phase 2 English and Scottish collections and observed similar separation of individuals of English and Scottish collection (Figure S1). In addition we used the Eigenvectors derived in the PCA of Phase 2 to compute projections of the components in Phase 1, thus checking that the components were representative of ancestry rather than a collection artefact (Figure S2).

We grouped Phase 2 individuals into ancestry clusters using the 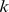-means clustering algorithm in the space defined by the top two leading principal components. As for Phase 1 individuals, the clustering with 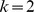 yielded the optimal BIC. This resulted in 21% of individuals changing label (Table S3). We then computed 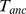, 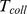 and D-statistics (as for Phase 1) and selected markers with D-statistics exceeding the 1% significance threshold (again, estimated from null simulations). Three markers passed through this filter. However, we observed consistent directions of effect across both Phases for only a single marker, rs10455 at the *CYBRD1* locus. At this marker we observed a genetic association with CRC in individuals from the English ancestry group (Phase 1 OR = 0.852, 95% CI = 0.736–0.986 p = 0.0321, 2-sided; Phase 2 OR = 0.904, 95% CI = 0.834–0.979] p = 0.0066, 1-sided), but no disease association for those in the Scottish ancestry group. Although, statistically, the evidence for heterogeneity and an English ancestry-specific disease association was mild and, indeed, we would expect exactly one marker to pass through the filters by chance, we proceeded with replication given the functional context of rs10455. rs10455 is located within the last exon of an iron-regulated gene, cytochrome B reductase 1, that is expressed in the intestine and plays a key role in dietary iron absorption (Genome Browser refSeq NM 001127383).

#### Replication stage

To ascertain independent evidence for the rs10455 association, we undertook a targeted replication stage in which we sought additional CRC case-control cohorts from equivalent (English and/or Scottish) populations. We identified a small Phase 3 multi-ethnic cohort (that is, mixture of English and Scottish collected individuals) of 631 cases and 731 controls. We did not have access to genome-wide genotype data and hence ancestry clustering could not be performed. However, as stated above, in the Replication step this is not prohibitive as the population label can be used as a noisy proxy for ancestry without biasing the result, albeit for a loss in power (see Text S1 and Tables S5 and S6).

Testing Phase 3 for variation in association signal for rs10455, using logistic regression, showed additional mild, but consistent evidence of a protective effect in England-collected individuals (OR = 0.886, 95% CI = 0.749–1.05, one-sided p = 0.083) and no evidence of association in the Scotland-collected Phase 3 individuals (OR = 1.09, 95% CI = 0.95–1.26, one-sided p = 0.899). Our power calculations (Table S5) suggested that the evidence of association in individuals of English ancestry was actually slightly stronger than expected under the evidence from Phases 1 and 2 of a 20% switch rate when ancestry was assigned and a true odds ratio of 0.9 (as estimated for rs10455 from the Phase 2 data).

Given the support for rs10455 in Phase 3, we undertook a further analysis and sought additional replication in a larger independent cohort of 7,395 cases and 4,202 controls collected in England (‘Phase 4’, Table S1). We supplemented Phase 4 with a further 5,193 Wellcome Trust Case Control Consortium 2 (WTCCC2) control samples known to be predominantly of English ancestry (2,692 from the 1958 British Birth Cohort and 2,501 from the UK National Blood Service, see Table S1 and Text S2). This sample size provided 99% power to detect the rs10455 association at the P = 0.05 level (assuming a 20% switch rate and the same OR). Given that we tested only a single marker, we need no multiple correction adjustment to the p-value for this Phase. We performed logistic regression analysis and confirmed association between CRC and rs10455 (OR = 0.933, 95% CI = 0.891–0.978, one-sided p = 0.00191).

**Figure 3:**
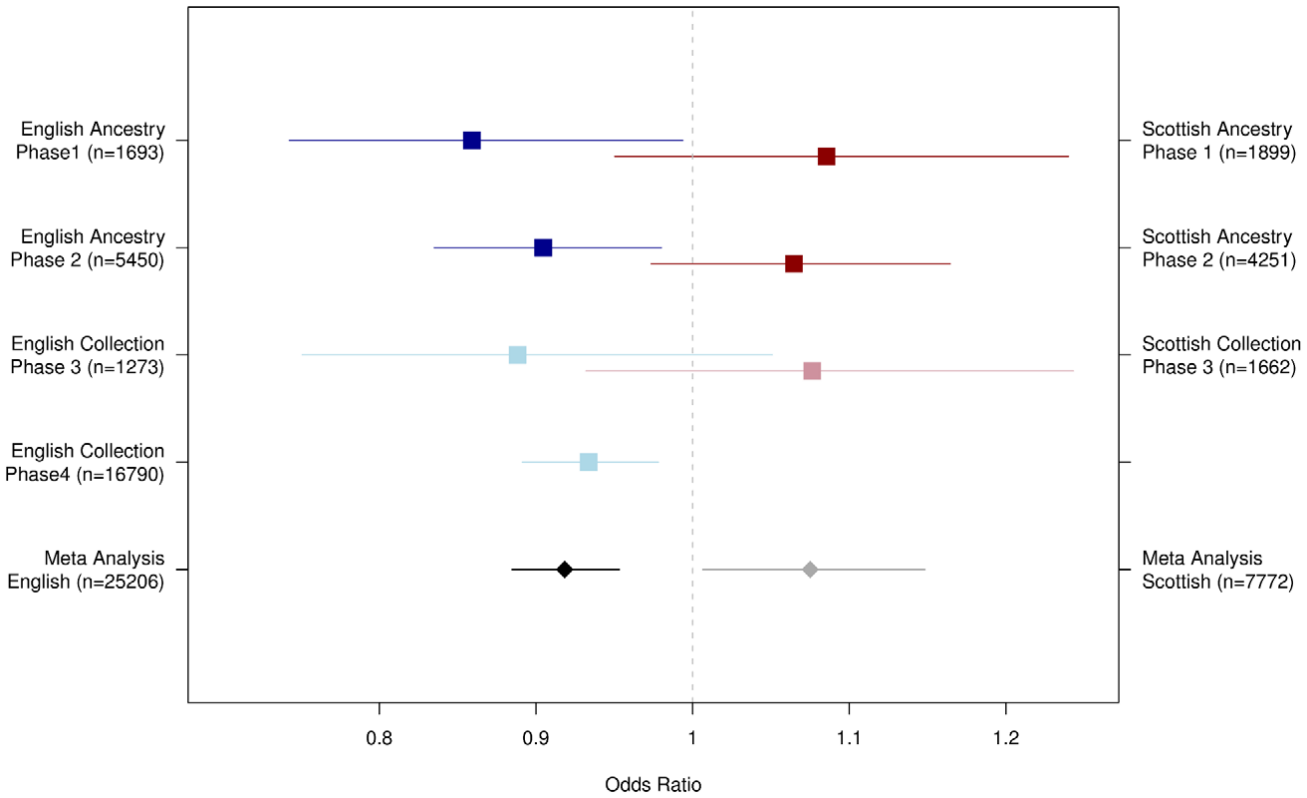
Summary of Association Evidence at rs10455. Forest plot of estimated effect sizes (with 95% confidence intervals). In Phases 1 and 2, the effect size is estimated from a logistic regression adjusted for gender within the English (blue) and Scottish (red) ancestry groups. In Phases 3 and 4 the effect size is estimated from a logistic regression adjusted for gender within the replication collection groups. The use of collections reduces power relative to what would be expected if ancestry groups were available. Finally we performed a meta-analysis combining all the individuals in Phase 1 and 2 Ancestry groups and Phase 3 and Phase 4 (English only) collection groups together with the additional WTCCC2 controls (coloured black and grey). The Odds Ratios are estimated from a logistic regression adjusted for gender.

Taken together, our analyses provide consistent evidence that variation at rs10455 (chr2:172,119,519 bases, NCBI Build 36), at the *CYBRD1* locus, associates with CRC risk in individuals of English ancestry, but not, at detectable levels, in those of Scottish ancestry (Figure 3 and Table S4). In a meta-analysis of the English samples from all Phases with adjustment for gender, we observed OR = 0.918 (95% CI 0.884–0.953) with one-side 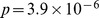. This is likely to be an underestimate of the true signal due the availability of English collection groups rather than ancestry groups in Phases 3 and 4.

The role of *CYBRD1* in iron absorption is of particular interest given that many studies have investigated the link between dietary iron intake and the risk of colorectal cancer. Although the relationship is not fully understood, increased dietary iron intake correlates with increased risk of colorectal tumours in several populations [20]–[23]. *CYBRD1* appears to bind quercetin which has been posited as a chemopreventive agent for colorectal cancer [24]. *CYBRD1* SNP rs884409 (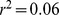, D′ = 1.0 with rs10455) has also been associated with serum ferritin levels [25] and *CYBRD1* is over-expressed in colorectal cancers [26]. Although rs10455 was originally genotyped as a tagSNP, it lies within the last exon of *CYBRD1*. This exon contains an alternative translation termination signal and rs10455 lies downstream of this signal, such that in one transcript it is a missense change, Ser266Asp. This change is predicted to be non-damaging by SIFT and Polyphen.

## Discussion

We have described a new method to detect interaction effects of specific genotypes on genetic background. It can be used to detect associations between genetic variants and disease that have been masked by population genetic heterogeneity. Our method can be extended easily to look for interactions on other covariates such as treatment or measured environmental exposures. We envisage our method being of particular interest to researchers with existing GWAS data sets, as the ancestry exposure variable can be easily defined and thus tested for interactions. Here we discuss particular issues that may arise in applying the method (Figure 1) to other study designs (for details of each step in the methodology see the *Materials and Methods* section).

### Single Collection

If only a single collection is available, the method can be applied by clustering on ancestry and investigating evidence by inspection of large 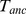 statistics and determining significance via null simulation; following only “Path A” of Figure 1 and steps 1, 2, 3, 5, 6. This will be at the expense of loss of power provided by the D-statistic filter.

### Non-overlapping Collections

In cases where there is near-complete confounding of collection and ancestry, our method can still be used to detect evidence of heterogeneity, but lacks the ability to distinguish whether the source is artifactual or the result of a disease risk factor correlating with ancestry. In such cases, evidence of heterogeneity would be assessed as for the Single Collection case using 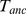 statistics (“Path A” of Figure 1 excluding step 4). Null simulations could be used to estimate thresholds for the T-statistics. If there are multiple independent collections from the same populations then artifacts can be ruled out by evidence of consistent results, and conventional meta-analysis approaches for heterogeneous effects could also be applied [27], although our approach should have increased power over this if the collections contain samples of mixed ancestry.

In selecting candidates for targeted replication, we suggest that both statistical evidence and functional context are considered. In our case, the statistical evidence for rs10455 was consistent but mild and indeed the marker may have been missed by a conventional GWAS analysis even within an English ancestry group. However, true interactions acting through say environmental effects covarying with ancestry may well lead to identified markers more closely related to disease mechanisms and hence located in or nearby genes with function previously implicated in disease processes, as was the case for *CYBRD1* here. For targeted replication where the ancestry (or original exposure) cannot be used directly, power calculations can be performed to show what level of evidence would be expected given a range of plausible switch/error rates, thereby guiding the sample sizes expected to be necessary. Details of power calculations are provided in the supplementary methods and implementation is included in the available R code.

It is important to note that statistical evidence of interaction of a polymorphism with an exposure need not reflect a direct biological interaction. Such evidence is also consistent for an interaction with an unobserved environmental exposure correlated with ancestry. For example, diet might correlate more strongly with ancestry than with residential location. Markers showing evidence of collection, but not ancestry, heterogeneity might also be of interest and could reflect any differences in the way the data was ascertained. This might result from technical artefacts or other sources of heterogeneity, such as those introduced by different ascertainment criteria.

Heterogeneity in ethnic origins or sample collection is usually regarded as a problem in GWAS owing to the type I and II errors that it can introduce into the analysis. We have shown, however, that heterogeneity between related populations can be used to a researchers's advantage when there is interaction between genotype and ethnicity, or a factor that co-varies with ethnicity. For such variants, our method potentially provides a way of identifying disease SNPs with relatively small effects that can be assessed further in appropriate validation sample sets without GWAS data or in other ways, such as functional evaluation. Such considerations apply to our putative colorectal cancer SNP rs10455 which demonstrated the potential of our technique for identifying interactions and hence predisposition genes.

Our clustering step makes discrete allocations of samples to clusters. It would be interesting to take into account the uncertainty in cluster membership within the association model, perhaps using a probabilistic clustering model, see for example [28]. This would be an interesting extension of the method we present and may further improve power to detect interactions.

## Materials and Methods

### Ethics Statement

The ethics board was the “Southampton and South-West Hampshire Research Ethics Committee (A)MREC/06\Q1702\99”. The collection of blood samples and clinico-pathological information from patients and controls was undertaken with written informed consent and ethical review board approval in accordance with the tenets of the Declaration of Helsinki.

### The Colorectal Cancer Cohorts

Table S1 provides detail about each of the CRC cohorts analysed in this paper. We have four phases of data; the first two were used for candidate discovery and the second two for replication.

#### Data used for Discovery

The discovery set of colorectal cancer cases and controls was derived from two distinct and independent collections, one based in Scotland only, and the other from across the UK, but predominantly from England. For convenience, we have analysed the Scottish collected Phase 1 in parallel with the UK collected Phase 1, and done the same for Phases 2.

Phase 1 samples (a total of 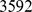) were genotyped at approximately 550,000 SNPs using the Illumina Hap550 or Hap300/Hap240S arrays. Based on SNPs with the best evidence of association (from the original GWAS [15]) in Scotland Phase 1 or UK Phase 1 or both (approximately equal number of SNPs in each category), 40,892 SNPs were then typed in 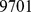 Phase 2 samples using a custom Illumina Infinium array.

Conventional GWAS data from these cohorts have been reported previously [13]–[16], [29]–[31].

#### Data used for Replication

Replication phase 3 consists of a total of 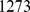 English collected individuals and 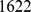 Scottish collected individuals [16]. Only a single marker (rs10455) was genotyped based on our Phase 1 and Phase 2 analyses for gene-ancestry interactions and thus collection label was used as a proxy for ancestry.

Replication phase 4 consists of 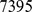 CRC cases and 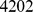 controls collected in solely in England and were genotyped at rs10455 only. In addition to boost power, we supplemented this phase with 5193 UK controls sampled as part of the Wellcome Trust Case-Control Consortium 2 (WTCCC2).

### Statistical Methods

The whole methodology is summarised by Figure 1. In this section each step is described fully.

#### Step 1: Defining the Ancestry and Collection Exposure Spaces

We performed a principal component analysis (PCA) to summarize observed relatedness of individuals. We selected markers for the PCA by first removing SNPs in regions of long-range linkage disequilibrium [17] and then by pruning the remaining markers to exclude markers short range LD (we used the sliding window method implemented in plink [32] with an 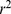 threshold of 0.2, a window width of 50 markers and a slide of 5 markers). We used the first two principal components to describe ancestry (in Phase 1 and 2 of our study) and checked they described ancestry rather than collection artifacts by projecting the components computed from Phase 2 samples in the Phase 1 samples. We observed similar separation of Scottish and UK collected samples as expected; more generally reference populations can be used to check for collection artifacts. At this stage we also note whether any of the principal components are significantly associated with phenotype so they can be included as adjustment variables in subsequent analyses.

The collection exposure is a one dimensional factor variable. It is fixed and defined according to design of the study/studies.

#### Step 2: Clustering Individuals by Ancestry and by Collection

We clustered individuals (in phase 1 and phase 2) in the space defined by the first two principal genetic components using the k-means algorithm [18]. We refer to these clusterings as ancestry clusterings and used the Bayesian information criterion (BIC) [19] to select the value of 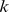, with the constraint that 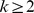. More generally any partitioning clustering algorithm can be used to cluster individuals in the space characterising genetic ancestry.

The collection clustering is defined by the collection factor variable and hence no clustering algorithm is required. Individuals of the same collection are clustered together with the number of clusters equal to the number of collection groups in the data. In our example there are two clusters, one containing individuals collected in Scotland and the other containing individuals collected predominantly in England.

In our analysis the number of principal components selected for defining the ancestry clustering was guided by differences observed between our collections. More generally, and if there is only a single cohort, this can be done by model-based variable selection such as the techniques implemented in R package *clustvarsel*. Other model-based clustering algorithms might be more appropriate for defining an ancestry clustering, particularly if the clusters are not of equal volume and shape as was the case with our example. We also note that the number of collection clusters need not be the same as the number of ancestry clusters; for example, there may be a single collection comprising multiple ancestry groups. Since the BIC is used for comparing models in our definition of the T- and D-Statistics (see *Materials and methods*), the number of clusters is automatically accounted for by our statistics. If the number of clusters is the same for both ancestry and collection groupings, it allows comparisons to be made easily and the switch rate from collection to ancestry can be determined, but this is not essential for the method to be effective.

#### Step 3A: Fitting the Heterogeneous Model of Association

To detect evidence of heterogeneous associations we fitted a mixture model for genetic association with phenotype. If multiple clusterings of the individuals in the space defining relatedness (or any other known source of heterogeneity) are considered then a mixture model is defined for each clustering (i.e. one for the Collection clustering and one for the Ancestry Clustering). For the general case, we let

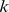
 be the number of clusters of individuals in the exposure/ancestry space.

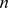
 be the total number of individuals in the study.

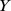
 be the 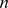 vector of phenotypes (binary for disease case-control studies).

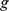
 be the 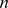 vector of genotypes (taking values 0,1,2 or missing).

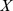
 be a matrix containing the exposure/ancestry variables. In our case for the ancestry exposure, 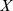 has dimension 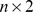 and contains the first and second principal genetic components for the individuals. More generally 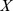 can contain 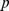 variables and can also define a non-genetic exposure.

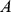
 be a matrix containing main effect adjustment covariates for all individuals. It has dimension 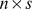 where 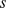 is the total number of adjustment covariates and entries 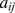 denote the values of the 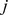
th covariate for the 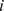
th individual. In our example, 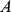 has two columns; one containing the gender indicator variable and the other the first principal genetic component (i.e. the first column of 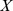).

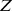
 be an indicator matrix containing cluster membership information. It is of dimension 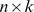 and for each individual 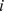, the entries 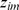 are binary and satisfy 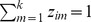 and
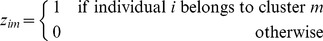

The 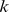 cluster model of heterogeneous association is then defined by the following generalised linear model:
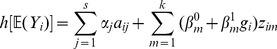
(1)


The function 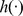 is the link function, 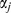 are adjustment parameters, 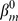 are cluster specific intercept terms and 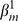 are the cluster specific genetic effect parameters. When 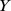 is a binary phenotype, the link function is taken as the logit function and the model is a logistic regression model. When 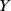 is a quantitative trait, the link function is typically the identity function and the model is an ordinary linear regression model.

The cluster specific intercept terms 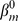 allow different clusters to have different background disease rates, while the cluster specific genetic effect terms 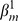 allows the marker being tested to have different associations to phenotype in different clusters. The model can be fitted to the data using the iterative re-weighted least squares algorithm to obtain maximum likelihood parameter estimates. Using these parameter estimates, we compute the log likelihood of the data under the fitted model and use this to define a measure of model fit. Since nested models with more parameters will always be more likely, we penalise the log likelihood (or more specifically minus twice the log likelihood) with a term that increases with the total number of parameters (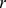) in the model. We compute the BIC which has penalty 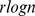. In this context 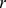 increases by two for each additional cluster (one extra intercept parameter and one extra genetic effect parameter). In the subsequent work we use 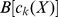 to denote the BIC score computed for the clustering 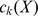 obtained by clustering individuals into 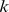 groups using exposure/ancestry variables 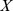.

#### Step 3B: Calculating T-statistics (T_anc_ and T_coll_)

To quantify evidence for the heterogeneous association we define the T-statistic by taking the difference in BIC statistics computed for the heterogeneous model and the null model of association thus accounting for model complexity.

To specify the null model, we note the null hypothesis is one of no interaction heterogeneity at the genetic marker. If this is true, there are two possible scenarios;

- There is no genetic association with phenotype for any of the individuals at the marker.
- There is a homogeneous genetic association with phenotype across all individuals at the marker.

In both of these instances it could also be there are different background prevalences of the disease and/or differences in allele frequency or linkage disequilibrium within different clusters of individuals (independently of genotype). It is important to allow for this in the specification of the null model to prevent within cluster main effects from dominating differences observed in the multi-cluster interaction model fit. Consequently the null model for the 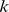 cluster alternative is
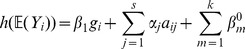



We compute the BIC statistic for the null model and use 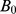
[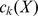] to denote this quantity. The 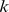-cluster null model contains 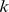
−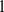 fewer parameters than the 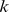-cluster heterogeneity model. It allows for different background prevalences of the disease within different clusters (i.e. the cluster specific intercept terms) and includes the ‘no association for any individuals’ scenario as a special case (i.e. 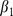 = 0). The distinction between scenarios (i) and (ii) can be made via assessment of the null model fit, however this is not our primary motivation. It is important to note that since our null model is one of homogeneity with respect to the genetic effect of a marker, our methods (by design) will not identify markers which were found to be associated with phenotype using homogeneous association testing techniques unless there is a considerable difference in the strength of associations between different clusters of individuals.

The test statistic (referred to as the T statistic) we use to assess evidence of heterogeneity is the difference in BIC for the heterogeneous and null models. If we are assessing the fit of a heterogeneous model with 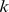 clusters, the test statistic is
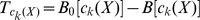
When the BIC is used for model selection, typically the model yielding the smallest BIC is selected, hence we might consider a positive BIC difference to be strong evidence of interaction heterogeneity. However, we use the difference in BIC as a test statistic and set significance thresholds by estimating its distribution under the null hypothesis.

In our analysis we refer to the 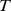 statistic computed using the ancestry clustering as 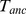 and refer to the 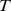 statistic computed using the collection clustering as 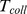.

#### Step 4: Calculating D-statistics

We have developed a statistical framework to compare two different exposure spaces (Ancestry and Collection in our work). More generally, we use 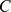 and 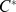 to denote two different clusterings of individuals in exposure spaces, denoted 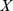 and 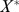 respectively. In our case, 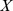 represents ancestry and is defined by the first two principal genetic components and 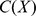 is the clustering of individuals in this space using two clusters. 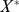 is the binary one-dimensional space indicating whether or not the individual was collected in England and 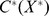 is the deterministic clustering imposed by the following indicator variable 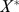 with entries
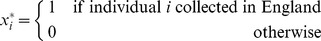



More generally, the exposure spaces 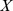 and 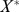 can be of different dimension and clusterings 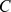 and 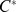 can have different values of 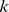 (in our case 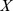 is two dimensional and 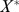 is one dimensional and both 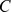 and 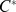 have 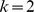 clusters). When 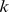 is the same for 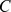 and 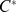, the degree of overlap between the clusterings can be quantified by the proportion of individuals that switch labels in one clustering with respect to the other. If one of the clusterings reflects the true source of the heterogeneity better than the other, then the increased error associated with the ‘worse’ clustering will lead to a weaker signal of interaction heterogeneity when our procedure is used. We quantify the difference in signal by computing the difference in test statistics:
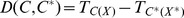
We refer to 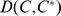 as the difference statistic. Positive values of the test statistic indicate that clustering 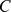 reflects the source of the interaction heterogeneity best, whereas negative values indicate that clustering 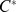 reflects the source of the interaction heterogeneity best. Given that one of the clusterings does reflect the true source of interaction heterogeneity, the magnitude of the difference in statistic will depend on the degree of overlap between the two clusterings and the strength of the associations. Small positive or negative difference statistics might reflect the fact that neither of the clusterings reflects the source of heterogeneity and indicate that evidence of interaction heterogeneity might have occurred by chance (although if clusterings have a high degree of overlap, difference statistics with small absolute values are likely to occur). In our analysis, the 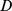-statistic computed compares the ancestry clustering with the collection clustering. Thus
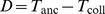



#### Step 5: Defining Significance Thresholds and Filtering Markers using evidence of Heterogeneity

There is no theoretical distribution for the D-Statistic, so we simulated null data sets (i.e. genotypes not associated with phenotype) to estimate the properties and quantiles of this distribution. Note that null distributions can differ between data sets according to the number of individuals in the data set, the number of clusters, and the contribution of adjustment variables to the model fit. The null hypothesis includes two scenarios. Firstly, no genetic association with phenotype for any individuals, and secondly, a homogeneous (non-zero) genetic association for all individuals. Since our method is designed to be used with GWA data, we assume that the vast majority of markers will fall into the first category. Consequently we estimate the distribution of test statistics under the null hypothesis assuming there is no genetic association with phenotype for any individuals. GWA case-control data sets are ascertained retrospectively, so we use a retrospective simulation technique i.e. simulating genotypes conditional on phenotype. The phenotype remains fixed (at the observed phenotype) for all simulations and the adjustment variables are also fixed and included in all interaction and null models. Since we assume there is no association of genotype with phenotype, under the assumption of Hardy-Weinberg equilibrium, the simulation of genotypes depends on the MAF. We investigated a range of allele frequencies from 0.05 to 0.5 by intervals of 0.05 and noted that the distribution of D-statistics were independent of MAF. Consequently to define a significance threshold for D-statistics we used the 99th centile of the statistics after pooling the 10
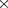
10,000 simulations across all allele frequencies. If only a single clustering is being tested for heterogeneity (and thus no difference statistics are calculated), thresholds on T statistics can be computed for each of the simulations instead.

A 99% one sided threshold for D-statistics is used to select markers enriched for ancestry heterogeneity rather than collection heterogeneity (i.e. exposure A relative to exposure B). To define a threshold to select markers enriched for collection heterogeneity (exposure B) the 1% centile could be chosen.

To identify candidate (interaction/heterogeneous) phenotype associated markers we suggest selecting markers whose D-statistics exceed the significance and then rank on the basis of the T statistic (in our case the ancestry T statistic T_anc_). If multiple phases of data are available for these markers then these markers can be checked for consistency across phases.

#### Step 6: Replication

Replication of candidate markers for interaction heterogeneity requires analysis of an independently collected data set, however it is important that the exposure(s) used to perform the original analysis can be collected (or estimated via an alternative, potentially surrogate source) and further more, that individuals in the replication phase lie in the same region of exposure space as the original data. Replication can also be undertaken even if the primary (ancestry) exposure and thus clustering cannot be computed directly provided a variable highly correlated with the primary exposure can be collected. The power to detect the interaction will be poorer (depending on the strength of the correlation between the proxy and the primary exposure) but power calculations can be undertaken to show the expected power.

In our example, in the replication phases 3 and 4 ancestry information was not available but we used geographical collection location as a proxy for it. We performed power calculations to show what we might expect to see given the expected error in using geographical collection location rather than ancestry. Details of our power calculations are documented in the Text S1 and can be modified for other data sets and/or exposure spaces.

## Supporting Information

Figure S1 **PCA Plot.** First principal component versus second principal component in Phase 2: Individuals in Phase 2 plotted in the space defined by the first two principal components computed from the genotype matrix. Individuals are coloured by their collection group and case-control status indicated by the plotting symbol.
(TIFF)

Figure S2 **PCA Projection.** Projection of Phase 1 genotypes onto Eigen vectors (defining the principal components) in Phase 2: Separation of the English and Scottish collected samples indicate the components were representative of ancestry rather than a collection artefact.
(TIFF)

Figure S3 **D-statistics.** The distribution of the D-Statistics computed for Phase 1. The 1% significance threshold is indicated by the red dashed line.
(TIFF)

Table S1 Cohorts details and genotype data collected at each phase.
(PDF)

Table S2 Composition of the ancestral clusters for phase 1 individuals stratified by collection source and disease status.
(PDF)

Table S3 Composition of the ancestral clusters for phase 2 individuals stratified by collection source and disease status.
(PDF)

Table S4 Summary of Evidence for CRC Association at rs10455 in English Collected/Ancestral Cohorts.
(PDF)

Table S5 Estimated power and p-values to detect an association at rs10455 in Phase 3 without principal component information as the switch rate varies.
(PDF)

Table S6 Estimated power and p-values to detect an association at rs10455 in Phase 4 without principal component information as the switch rate varies.
(PDF)

Text S1 Description of Power Calculations for Replication Phases 3 and 4.
(PDF)

Text S2 Web Resources.
(PDF)
