# Supplementary figures and images for: A Novel Test for Gene-Ancestry Interactions in Genome-Wide Association Data
Source: PLoS One. 2012 Dec 6;7(12):e48687. doi: 10.1371/journal.pone.0048687 (PMC3516524; doi:10.1371/journal.pone.0048687)

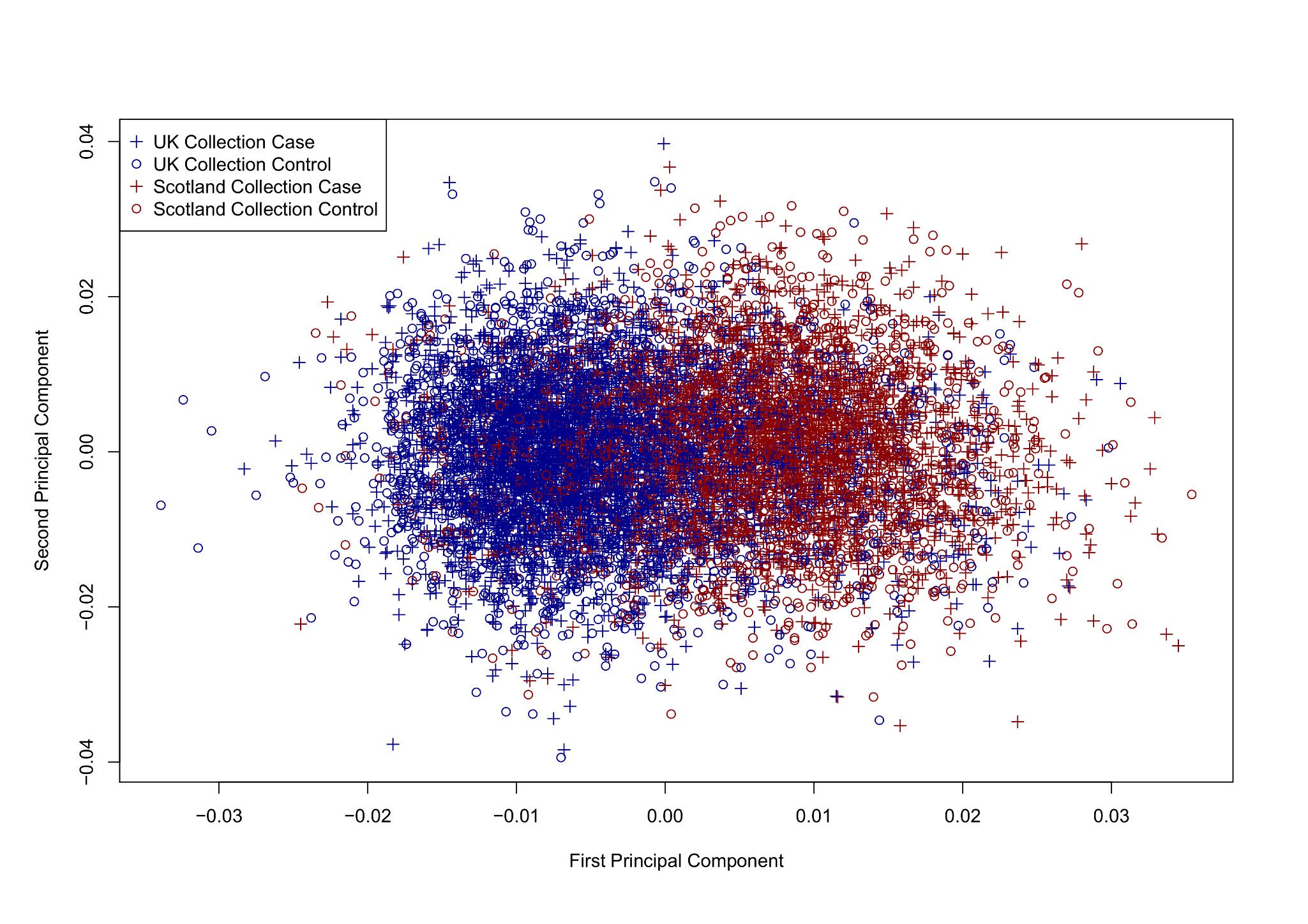

Supplement: Figure S1 — PCA Plot. First principal component versus second principal component in Phase 2: Individuals in Phase 2 plotted in the space defined by the first two principal components computed from the genotype matrix. Individuals are coloured by their collection group and case-control status indicated by the plotting symbol. (TIFF) [file pone.0048687.s001.tiff]

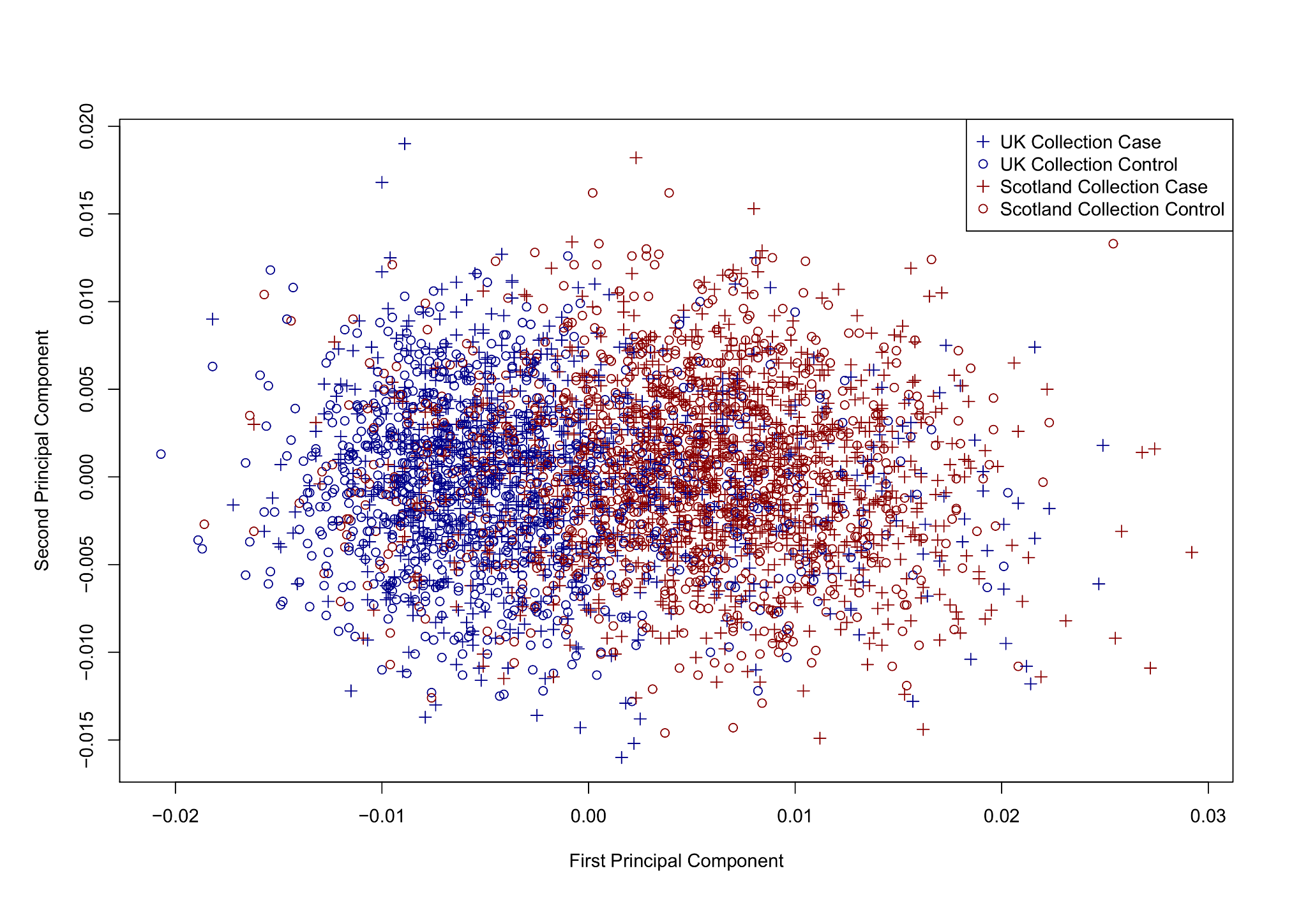

Supplement: Figure S2 — PCA Projection. Projection of Phase 1 genotypes onto Eigen vectors (defining the principal components) in Phase 2: Separation of the English and Scottish collected samples indicate the components were representative of ancestry rather than a collection artefact. (TIFF) [file pone.0048687.s002.tiff]

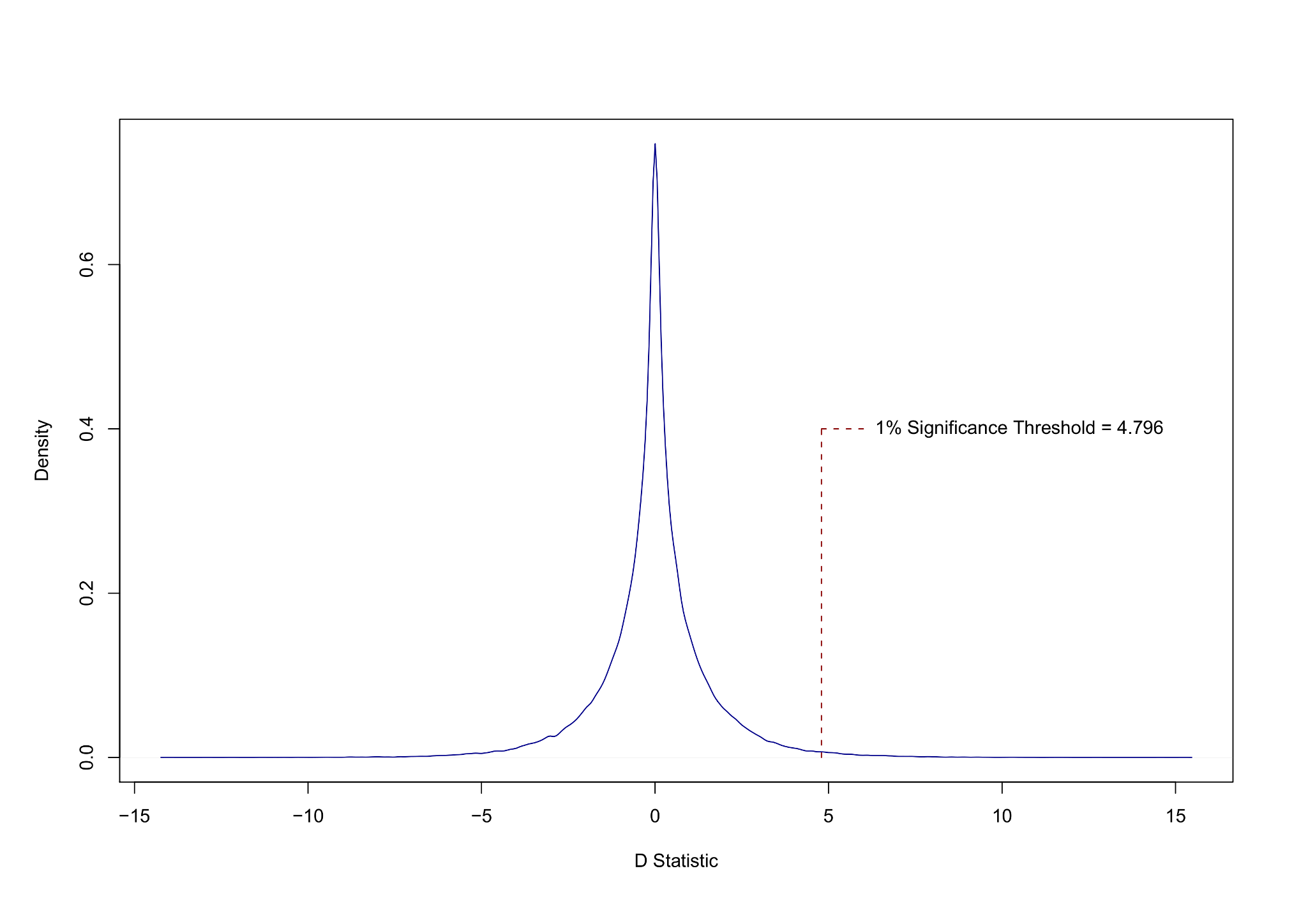

Supplement: Figure S3 — D-statistics. The distribution of the D-Statistics computed for Phase 1. The 1% significance threshold is indicated by the red dashed line. (TIFF) [file pone.0048687.s003.tiff]
