# Supplementary material for: A Novel Test for Gene-Ancestry Interactions in Genome-Wide Association Data
Source: PLoS One. 2012 Dec 6;7(12):e48687. doi: 10.1371/journal.pone.0048687 (PMC3516524; doi:10.1371/journal.pone.0048687)
Supplement: Table S1 — Cohorts details and genotype data collected at each phase. (PDF) [file pone.0048687.s004.pdf]

Table S1. Cohorts details and genotype data collected at each phase.

Phase	Cohort	Collection	Recruitment Criteria		Number of subjects		Age range		Genotyping
		**Origin**	Cases	Controls	Cases	Controls	Cases	Controls	**Platform**
Phase 1	CORGI	UK	Diagnosed CRC, CR adenoma or, large aggressive adenoma	Unaffected partners (no family history)	922	927	≤ 75	≤ 75	Illumina Hap550
Phase 1	COGS	Scotland	Diagnosed CRC	Matched random sample (age,sex)	980	1002	≤ 55	≤ 60	Illumina Hap300 Hap240
Phase 2	NSCCG	UK	Diagnosed CRC	Unaffected partners (no malignancies or family history)	2873	1235	≤ 70	≤ 75	Illumina custom array (40,829 markers)
	GELCAPS	UK	-		-	917	-	≤ 70	
	RMHNHST	UK	-		-	719	-	≤ 70	
Phase 2	SOCCS	Scotland	Diagnosed CRC	Cancer free matched random sample (age, sex residential area)	2023	2092	≤ 85	≤ 85	Illumina custom array (40,829) markers)
Phase 3	CORGI (2)	UK	as CORGI	as CORGI	631	567	≤ 75	≤ 75	KASPar (rs10455)
	Notts	UK	-	random pop sample	-	164	-	≤ 30	
Phase 3	SOCCS (2)	Scotland	as SOCCS	Cancer-free pop sample	940	975	≤ 85	≤ 85	KASPar (rs10455)
Phase 4	NSCCG (2)	England	as Phase 2	as Phase 2	7395	4202	≤ 70	≤ 75	KASPar (rs10455)
	NBS	England	-	Random sample UK blood donors	-	2501	-	≤ 69	Illumina custom array
	BC58	England	-	British sample born in 1958	-	2692	-	53	Illumina custom array

CORGI: Colorectal Tumour Gene Identification Consortium

NSCCG: National Study of Colorectal Cancer Genetics

RMHNHST: Royal Marsden Hospital NHS Trust

GELCAPS: Genetic Lung Cancer Predisposition Study

COGS: Colorectal Cancer Genetics Study

SOCCS: Scottish Colorectal Cancer Study

NBS: UK National Blood Service Cohort (WTCCC2)

BC58 1958 British Birth Cohort (WTCCC2)
