# Supplementary material for: A Novel Test for Gene-Ancestry Interactions in Genome-Wide Association Data
Source: PLoS One. 2012 Dec 6;7(12):e48687. doi: 10.1371/journal.pone.0048687 (PMC3516524; doi:10.1371/journal.pone.0048687)
Supplement: Table S2 — Composition of the ancestral clusters for phase 1 individuals stratified by collection source and disease status. (PDF) [file pone.0048687.s005.pdf]

Table S2. Composition of the ancestral clusters for phase 1 individuals stratified by collection source and disease status.

	**Scottish Ancestry (cases : controls)**	**English Ancestry (cases : controls)**	**Total (cases : controls)**
**Scotland Collection**	**1579** (787 : 792)	347 (168 : 179)	1926 (955 : 971)
**UK Collection**	320 (174 : 146)	**1346** (643 : 703)	1666 (817 : 849)
**Total**	1899 (961 : 938)	1693 (811 : 882)	**3592** (1772 : 1820)
