# Supplementary material for: A Novel Test for Gene-Ancestry Interactions in Genome-Wide Association Data
Source: PLoS One. 2012 Dec 6;7(12):e48687. doi: 10.1371/journal.pone.0048687 (PMC3516524; doi:10.1371/journal.pone.0048687)
Supplement: Table S3 — Composition of the ancestral clusters for phase 2 individuals stratified by collection source and disease status. (PDF) [file pone.0048687.s006.pdf]

Table S3. Composition of the ancestral clusters for phase 2 individuals stratified by collection source and disease status.

	**Scottish Ancestry (cases : controls)**	**English Ancestry (cases : controls)**	**Total (cases : controls)**
**Scotland Collection**	**3122** (1597 : 1525)	913 (395 : 518)	4035 (1992 : 2043)
**UK Collection**	1129 (570 : 559)	**4537** (2267 : 2270)	5666 (2837 : 2829)
**Total**	4251 (2167 : 2084)	5450 (2662 : 2788)	**9701** (4829 : 4872)
