# Supplementary material for: A Novel Test for Gene-Ancestry Interactions in Genome-Wide Association Data
Source: PLoS One. 2012 Dec 6;7(12):e48687. doi: 10.1371/journal.pone.0048687 (PMC3516524; doi:10.1371/journal.pone.0048687)
Supplement: Table S4 — Summary of Evidence for CRC Association at rs10455 in English Collected/Ancestral Cohorts. (PDF) [file pone.0048687.s007.pdf]

**Table S4.** Summary of Evidence for CRC Association at rs10455 in English Collected/Ancestral Cohorts.

| Phase | OR (95% CI) | Logistic Regression (gender adjusted) p-value | N Cases | N Controls | Classification |
|---|---|---|---|---|---|
| 1 | 0.859 (0.742-0.994) | 0.04090 (two-sided) | 811 | 882 | English Ancestry |
| 2 | 0.905 (0.835-0.980) | 0.00714 (one-sided) | 2662 | 2788 | English Ancestry |
| 3 | 0.888 (0.751-1.051) | 0.08357 (one-sided) | 581 | 692 | English Collection |
| 4 | 0.933 (0.891-0.978) | 0.00191 (one-sided) | 7395 | 9395 | English Collection |
| Meta | 0.918 (0.884-0.953) | $3.9 \times 10^{-6}$ (one-sided) | 11449 | 13757 | Mixture |
