# Supplementary material for: A Novel Test for Gene-Ancestry Interactions in Genome-Wide Association Data
Source: PLoS One. 2012 Dec 6;7(12):e48687. doi: 10.1371/journal.pone.0048687 (PMC3516524; doi:10.1371/journal.pone.0048687)
Supplement: Table S6 — Estimated power and p-values to detect an association at rs10455 in Phase 4 without principal component information as the switch rate varies. (PDF) [file pone.0048687.s009.pdf]

Table S6. Estimated power and p-values to detect an association at rs10455 in Phase 4 without principal component information as the switch rate varies. Estimates for each switch rate were calculated on the basis of 10,000 simulations (simulated data sets described in the supplementary text). 'Average P' is the average one-sided p-value for the logistic regression of the disease status on the (simulated) genotype and 'Power' is the estimated power to detect a disease association using a logistic regression with one-sided $p < 0.05$. At each switch rate, power is estimated by the proportion of simulated data sets yielding $p < 0.05$ for the logistic regression of disease status on (simulated) genotype.

**Switch Rate**	**Average P**	**Power (p<0.05)**
0.00	0.00431	0.9820
0.05	0.00641	0.9696
0.10	0.00921	0.9531
0.15	0.01318	0.9318
0.20	0.01822	0.9082
0.25	0.02443	0.8735
