# Supplementary material for: A Novel Test for Gene-Ancestry Interactions in Genome-Wide Association Data
Source: PLoS One. 2012 Dec 6;7(12):e48687. doi: 10.1371/journal.pone.0048687 (PMC3516524; doi:10.1371/journal.pone.0048687)
Supplement: Text S1 — Description of Power Calculations for Replication Phases 3 and 4. (PDF) [file pone.0048687.s010.pdf]

Text S1. Description of Power Calculations for Replication Phases 3 and 4.

In phase 3 and phase 4 we looked for evidence of a gene interaction with collection group rather than ancestry group since we cannot perform a PCA without genome-wide data. In particular we looked for evidence of an association between rs10455 and CRC risk in the English collected groups and look for no association in Scottish collected group. The lack of a PCA reduces power due to the error incurred by using collection groups rather than ancestry groups but does not introduce upward bias. We investigated the loss of power from the associations we would expect to observe given the estimated effect size of rs10455 (from the phase 2 English ancestry group) across a range of error rates (i.e. the percentage of individuals who would switch label between ancestry and collection clusters). In phase 1 and phase 2 the error rates were 0.185 and 0.210 respectively so we simulated errors in the range 0-0.25. We performed simulations for the English collections of phase 3 and phase 4 separately using the observed sample sizes (Tables A and B in this document). At each simulation, we simulated a set of genotypes conditional on the phenotype and the observed (i.e. the estimated) effect at rs10455. We fitted a logistic regression for disease association at the simulated genotypes and recorded the one-sided p-values of disease association.

Our simulations showed we have $< 40\%$ power to detect an association at the 0.05 significance level in phase 3 due to small sample size, but by computing average p-values (for simulations at each error rate), we estimated the degree of evidence we would expect to see in this sample (Supplementary Table S9). Our observed p-value in the phase 3 English collection is smaller than we might expect even with no switching (observed p = 0.083, expected p $\approx$0.2). In phase 4, we estimated we have between 87-98% power to detect an association for error rate between zero and 0.25 (Supplementary Table S10) and our observed p-value is consistent with what we might expect for a switch rate in the range 15-20%.

All simulations were performed using R code. It is available at request from the corresponding author.
