# Supplementary material for: A Novel Test for Gene-Ancestry Interactions in Genome-Wide Association Data
Source: PLoS One. 2012 Dec 6;7(12):e48687. doi: 10.1371/journal.pone.0048687 (PMC3516524; doi:10.1371/journal.pone.0048687)
Supplement: Text S2 — Web Resources. (PDF) [file pone.0048687.s011.pdf]

Text S2. Web Resources.

Plink: http://pngu.mgh.harvard.edu/ purcell/plink/

R: http://cran.r-project.org/

WTCCC2: https://www.wtccc.org.uk/ccc2/

Genome Browser: http://genome.ucsc.edu/

SIFT: http://sift.jcvi.org/

PolyPhen: http://genetics.bwh.harvard.edu/pph/
